# Microbially induced corrosion of carbon steel in deep groundwater environment

**DOI:** 10.3389/fmicb.2015.00647

**Published:** 2015-07-24

**Authors:** Pauliina Rajala, Leena Carpén, Mikko Vepsäläinen, Mari Raulio, Elina Sohlberg, Malin Bomberg

**Affiliations:** ^1^Materials Performance, VTT Technical Research Centre of FinlandEspoo, Finland; ^2^CSIRO Mineral Resources Flagship, MelbourneVIC, Australia; ^3^Geobiotechnology, VTT Technical Research Centre of FinlandEspoo, Finland

**Keywords:** deep biosphere, microbial corrosion, terrestrial biosphere, carbon steel, sulfate reducing bacteria

## Abstract

The metallic low and intermediate level radioactive waste generally consists of carbon steel and stainless steels. The corrosion rate of carbon steel in deep groundwater is typically low, unless the water is very acidic or microbial activity in the environment is high. Therefore, the assessment of microbially induced corrosion of carbon steel in deep bedrock environment has become important for evaluating the safety of disposal of radioactive waste. Here we studied the corrosion inducing ability of indigenous microbial community from a deep bedrock aquifer. Carbon steel coupons were exposed to anoxic groundwater from repository site 100 m depth (Olkiluoto, Finland) for periods of 3 and 8 months. The experiments were conducted at both *in situ* temperature and room temperature to investigate the response of microbial population to elevated temperature. Our results demonstrate that microorganisms from the deep bedrock aquifer benefit from carbon steel introduced to the nutrient poor anoxic deep groundwater environment. In the groundwater incubated with carbon steel the planktonic microbial community was more diverse and 100-fold more abundant compared to the environment without carbon steel. The betaproteobacteria were the most dominant bacterial class in all samples where carbon steel was present, whereas in groundwater incubated without carbon steel the microbial community had clearly less diversity. Microorganisms induced pitting corrosion and were found to cluster inside the corrosion pits. Temperature had an effect on the species composition of microbial community and also affected the corrosion deposits layer formed on the surface of carbon steel.

## Introduction

During the last two decades the Fennoscandian deep terrestrial biosphere has been extensively studied due to the geological repositories planned for spent nuclear fuel and radioactive decommissioning waste (Haveman and Pedersen, 2002; Itävaara et al., 2011; Purkamo et al., 2013; Bomberg et al., 2015; Rajala et al., 2015). Despite the extensive study of deep biosphere, the potential of indigenous microbial communities to induce corrosion under the prevailing environmental conditions is not well known. The assessment of microbially induced corrosion (MIC) of carbon steel in deep bedrock environment has become important for evaluating the long-term safety of disposal of low and intermediate level radioactive (LLW/ILW) waste. Such waste is produced during the operation, maintenance, and decommissioning of nuclear power plants (NPPs). The LLW/ILW produced in Olkiluoto NPP, Finland, has since 1992 been disposed into an underground repository excavated in to bedrock to a depth of 60–100 m below sea level [Teollisuuden voima Oyj (TVO), 2015]. The metallic portion of LLW/ILW generally consists of carbon steel and stainless steels.

In oxygen-free water, the corrosion rate of carbon steel is low, unless the water is acidic or microbial activity in the environment is high. In Olkiluoto NPP the LLW/ILW is packed into concrete silos or concrete boxes. Concrete generates a high pH environment, which in turn is assumed to reduce the corrosion rate of carbon steel (Rajala et al., 2014). Nevertheless, over the time the pH decreases, for example due to the carbonization of concrete.

The groundwater at the repository depth contains large microbial populations of up to 10^5^ microorganisms mL^-1^ with considerable diversity of species (Haveman and Pedersen, 2002; Nyyssönen et al., 2012). Microorganisms may significantly contribute to the corrosion of metallic waste and can accelerate corrosion rates up to 1000–10,000 fold (Videla, 1996; Carpén et al., 2013). Microorganisms are able to affect various corrosion mechanisms, such as general corrosion and localized corrosion, the latter including pitting and stress corrosion cracking (Little et al., 1992). Sulfate-reducing bacteria (SRB), in particular, are known to cause corrosion of carbon steel (Bryant et al., 1991). Carpén et al. (2012) demonstrated that the corrosion rate might be as high as 10–63 μm a^-1^ in the repository environment and localized corrosion rates even higher. Such high corrosion rates in anoxic groundwater cannot occur without acceleration of the corrosion process by microbial activity. Especially microbial biofilm formed on carbon steel surface has been shown to accelerate corrosion (Little et al., 1992).

In nutrient poor conditions, such as in deep anoxic groundwater, microorganisms may significantly accelerate the corrosion rate of metals by using metallic iron as electron donor (Xu and Gu, 2011). Microorganisms are also able to produce corrosive agents, such as organic acids or sulfide, and consume hydrogen that is produced in the corrosion process (Little et al., 1992; Lee et al., 1995). As a consequence of the accelerated corrosion and increased solubility of the metal ions in acidic environments, radioactive nuclides may be released into groundwater and transferred to neighboring areas of the repository.

The aim of this study was to characterize microbial biofilms associated with corrosion, study the ability of the indigenous groundwater microbes to benefit from carbon steel, and compare the corrosion rate of carbon steel in groundwater at room temperature and *in situ* temperature. In general, elevated temperatures accelerate corrosion, but the effect of temperature on the corrosion-accelerating bacterial community is unknown. Electrochemical measurements are often conducted at room temperature instead of at *in situ* temperature. Therefore, the effect of temperature on the community composition of the biofilm formed on the surface of carbon steel was also studied.

## Materials and Methods

### Sample Collection

The experiment was conducted using the native groundwater from Olkiluoto VLJ-cave the geological disposal site of low and intermediate level radioactive waste (LLW/ILW) (61°14′13″N 21°26′27″E) from the drill hole located next to the repository silos. The drill hole was located at the depth of 100 m, which is the same level as the bottom of the repository silo and can be reached from the maintenance tunnel in the repository cave. Groundwater sampling was performed in June 2011. The groundwater was funneled into an anoxic glove bag (AtmosBag^®^, Sigma-Aldrich, St. Louis, MO, USA) without exposure to oxygen through a factory clean polyamide tube directly from the drill hole located in the cave wall. Anoxic conditions within the glove bag were maintained with constant N_2_ (99.999%) flow and anaerobic generators (Anaerocult^®^ A, Merck, Germany). In the anoxic glove bag, microbial biomass for DNA extraction was collected from three parallel 250 mL groundwater samples on 0.22 μm pore-size Sterivex- filtration units (Millipore, Billerica, MA, USA) for DNA extraction. The filtration units were frozen on dry ice immediately after sampling.

Sterile, acid washed, 250 mL boro-silicate incubation bottles (Schott, Germany) each containing one carbon steel coupon (AISI/SAE 1005, 5 mm × 80 mm × 1 mm; **Table 1**) had been prepared in advance by autoclaving. The bottles were rendered anoxic under N_2_ flow inside the glove bag on site. The surface finish of carbon steel coupons was as received. The incubation bottles were filled with 250 mL groundwater and were subsequently sealed with tight fitting butyl rubber septum and open top cap within the glove bag to enable anoxic sampling from the incubation bottles. The carbon steel coupons were exposed to groundwater in the microcosms at room temperature or at 6°C, which represents *in situ* temperature of the repository cave, for 3 and 8 months. In addition, reference groundwater incubations without carbon steel coupons were prepared in order to observe the change in the microbial community during the incubation without the influence of carbon steel. Three replicate groundwater samples were prepared for microbiological analyses and corrosion analyses of each treatment, respectively.

**Table 1:** Composition of carbon steel.

C	Si	Mn	S	P	Cr	Ni	Mo	Cu	Al	W	V	Ti	Co	B
0.03	0.009	0.21	0.007	0.007	0.04	0.05	0.01	0.02	0.048	<0.01	0.003	0.002	0.004	–

### Groundwater Chemistry

The conductivity (Radiometer CDM92, Radiometer, France) and pH (HACH Sension 156, Loveland, CO, USA) of the groundwater was measured immediately on site. The chemical composition of the groundwater was analyzed at the TVO’s laboratory (Eurajoki, Finland). At the end of the incubation periods (3 and 8 months) the pH, conductivity, redox-potential and oxygen content of the water in each bottle were measured inside the glove bag to protect the samples from oxygen. Conductivity was measured as above, pH using a Orion 5-star pH meter, redox with a HACH Sension 156 meter and oxygen with a dissolved oxygen test kit (CHEmetrics, 0–1 ppm, Midland, VA, USA; 3 months exposure) or HACH Sension 156 m (8 months exposure).

### Nucleic Acid Extraction

After the incubation periods (3 and 8 months) the carbon steel coupons were removed from the bottles under N_2_ flow inside the glove bag. Coupons for molecular biological analyses (three parallel coupons for each treatment) were stored in sterile 50 mL screw cap test tubes (Corning, Tewksbury, MA, USA) at -80°C. The microbial biomass from the water from each incubation bottle was collected on Sterivex- filtration units by filtration, and they were frozen at -80°C until DNA extraction. DNA was extracted from microbial biomass collected on filtration units from 3 replicate water samples. The biofilm DNA was extracted from the surface of the carbon steel as described by Rajala et al. (2014). Briefly, the carbon steel coupons were shaken in 10 mL sterile phosphate buffered saline (PBS) and Tween^®^20 (Merck, Germany; 1 μL Tween^®^20 1 mL^-1^ PBS) for 20 min at 150 rpm agitation followed by ultra sonication for 3 min. The removed biomass was then collected on Sterivex- filtration units. For DNA extraction, the filtration units were opened in a laminar flow hood with flame-sterilized pliers as described by Itävaara et al. (2011). DNA was extracted using the PowerWater DNA Isolation kit (MoBio Laboratories, Inc., Carlsbad, CA, USA) in accordance with the manufacturer’s protocol and eluted in 50 μL elution buffer supplied by manufacturer. Possible contamination from different reagents and sample handling was determined by including DNA extraction controls. These controls comprised unused Sterivex- filtration units and the same DNA extraction protocol as used for the samples. These negative reagent control extraction were performed in parallel with the DNA extraction of the actual samples.

### Quantitative PCR

Quantitative PCR (qPCR) was used to determine the abundance of bacteria and sulfate reducers based on the amount of 16S rRNA and *dsr*B gene copies in the groundwater and in the biofilm. qPCR was performed in 10 μL reaction volumes using LightCycler 480 qPCR instrument and LightCycler 480 Software 1.5.0 (Roche Applied Science, Germany). The reaction mixture contained 1 μL DNA template, standard dilution or water, 1 × KAPA SYBR^®^ FAST Universal qPCR Master Mix (KAPA Biosystems, Wilmington, MA, USA), 2.5 μM of both forward and reverse primer and nuclease free water. The primers used for the bacterial 16S rRNA gene qPCR were fD1 (Weisburg et al., 1991) and P2 (Muyzer et al., 1993) and for *dsr*B genes primers 2060F and dsr4R (Wagner et al., 1998; Geets et al., 2006). As a standard, a plasmid dilution series containing the corresponding gene insert (10^1^–10^9^ copies per reaction) was used. The PCR program consisted of an initial 15 min incubation at 95°C, followed by 45 cycles of denaturation at 95°C for 10 s, annealing at 55°C for 35 s and extension 72°C for 30 s, and with final extension at 72°C for 3 min. Sample fluorescence was measured at the end of each elongation phase. Subsequently, a melting curve was recorded, to test the specificity of the qPCR, with a program consisting of 10 s of denaturation at 95°C, 1 min of annealing at 65°C, and a melting and continuous measuring step rising gradually (20°C s^-1^) to 95°C. Three parallel qPCR reactions were performed for each extracted DNA sample, as well as for the no-template controls which were used to determine the background noise of the qPCR.

One-way analysis of variance and subsequent multiple comparisons analysis using Tuckey’s variance test was performed for qPCR results using the PAST software version 3.06 (Hammer et al., 2001).

### DGGE Analysis of the Bacterial and Sulfate Reducing Community

The bacterial diversity was investigated by PCR-denaturing gradient gel electrophoresis (DGGE) analysis of 16S rRNA gene fragment and SRB diversity was investigated by PCR-DGGE analysis of the dissimilatory sulphite reductase gene (*dsr*B) fragment as described in Muyzer et al. (1993) and Nyyssönen et al. (2012), respectively. For the 16S rRNA gene targeted DGGE, a 193 bp fragment covering the V3 variable region of the bacterial 16S rRNA gene was amplified with primers P2 and P3 (Muyzer et al., 1993). The amplification was performed in 50 μL reaction volumes, containing 4 μL template or nuclease free H_2_O for no template controls, 1 × Dynazyme Reaction Buffer (Finnzymes, Finland), dNTP 62.5 μM each (final concentration; Finnzymes, Finland), 0.2 μM (final concentration) of both forward- and reverse primers (Eurogentec, Sweden), 1 U Dynazyme II polymerase (Finnzymes, Finland) and nuclease free H_2_O (Sigma, St. Louis, MO, USA). Formamide, 0.1 μL 10^-1^ μL reaction mix, was used for amplification of the 16S rRNA gene fragments. The amplification in all PCR reactions was carried out on a thermal PCR cycler (Mastercycler gradient, Eppendorf, Germany) using the following conditions: 95°C initial denaturation for 5 min, followed by 40 cycles of 94°C for 1 min, 55°C for 1 min and 72°C for 1 min, with a final extension step at 72°C for 10 min. PCR products were visualized with agarose gel electrophoresis on a 1% agarose gel (LE-agarose, Lonza, Switzerland) in 1 × SB-buffer (Brody and Kern, 2004). The gel was stained with 1 × SyberSafe (Invitrogen, Carlsbad, CA, USA) and run at 300 V for 15 min. The 16S PCR products were resolved by DGGE on 8% acrylamide with denaturing gradient 20–65% at constant voltage of 65 V and temperature at 60°C for 18 h in 0.5 × TAE running buffer as described by Itävaara et al. (2011).

A 470 bp fragment of the *dsrB* gene was amplified with primers 2060F+GC and dsr4R (Wagner et al., 1998; Geets et al., 2006). The PCR program and reaction mixture were as described above. The *dsr*B PCR products were resolved with a denaturing gradient of 40–70% at constant voltage of 85 V and temperature at 60°C for 20 h in 0.5 × TAE running buffer. The gels were imaged and DNA from DGGE bands was prepared for sequencing as described by Nyyssönen et al. (2012).

The DNA fragments were sequenced at Macrogen Inc. (Korea) using primer P2 and dsr4R primer for the 16S rRNA and *dsr*B gene fragments, respectively.

### Analysis of DGGE Profiles and Phylogeny

The obtained DGGE profiles were normalized and analyzed with the BioNumerics software version 5.10 (Applied Maths, Kortrijk, Belgium). During this processing, the different DGGE lanes were defined, background was subtracted, differences in the intensity of the lanes were compensated by normalization, and a correlation matrix was calculated. Clustering was done with Dice’s coefficient for similarity.

Sequences obtained from DGGE bands were imported into the Geneious Pro Software package (version 5.5.6, Biomatters Inc., New Zealand, Drummond et al., 2010) where they were manually checked, assembled and edited before subjecting them to phylogenetic analyses. The sequences were compared with the BLAST tool (blastn and blastx, http://blast.ncbi.nlm.nih.gov/Blast.cgi; Altschul et al., 1990) to the sequences in NCBI databases. The closest matching sequences, relevant reference sequences and sequences of type strains were included in the phylogenetic analyses. The nucleic acid sequences of the bacterial 16S rRNA gene fragments were aligned using ClustalW (Thompson et al., 1994) in Geneious Pro, and the alignment was manually edited. The *dsr*B sequences were converted to amino acid sequences and aligned using ClustalW using default parameters. The alignments were checked and manually edited. Maximum likelihood analysis was performed using PhyML (Guidon and Gascuel, 2003) with the Jukes–Cantor substitution model (Jukes and Cantor, 1969) for the nucleic acid sequence alignments, and the Whelan and Goldman (WAG) substitution model (Whelan and Goldman, 2001) for the amino acid sequence alignments. Bootstrap support for the nodes was calculated on 1000 random repeats. Due to the high number of similar 16S rRNA and *dsr*B gene sequences, the sequences were grouped into operational taxonomic units (OTUs) according to their alignment. Each OTU consisted of 16S rRNA or *dsr*B sequences sharing over 97% similarity of the nucleic acid or amino acid sequence, respectively. One representative sequences was chosen from each sample for each phylogenetic group.

### Accession Numbers

The obtained sequences (16S rRNA, *dsr*B) have been submitted to European Nucleotide Archive (ENA), and are available under accession numbers LN869402–LN869518.

### Corrosion

After retrieving the carbon steel coupons for corrosion analyses they were immediately rinsed with ethanol (96%) and air-dried and stored in glass desiccators until analyzed. The representative coupons from both temperature treatments (RT and 6°C) were chosen for examination with an energy-dispersive x-ray spectrometry (EDS) coupled to scanning electron microscopy (SEM). The coupons were weighed, cleaned with a brush and pickled according to the Standard Practice for Preparing, Cleaning, and Evaluating Corrosion Test Coupons (ASTM standard G 1-90, 2011). The pickling (HCl + 20 g/L Sb_2_O_3_ + 50 g/L SnCl_2_, 23°C, 5 min) was performed four times. To determine the mass loss of the base metal when removing the corrosion products, a replicate uncorroded control coupon was cleaned by the same procedure as that used for the test coupons. Loss of mass of the carbon steel coupons was determined and the average corrosion rates (μm a^-1^) were calculated in the following way (Equation 1):

(1) Corrosionrate = (K × W)/(A × T × D),

where K, constant (0.365 × 10^4^), W, mass loss (mg), T, time of exposure (days), A, area (cm^2^) of carbon steel coupon, D, density (carbon steel)(g/cm^3^).

The coupons were subsequently inspected under a low magnification stereomicroscope to reveal the nature of the possible corrosion attack.

### Scanning Electron Microscopy of Biofilms

Carbon steel coupons were immersed in phosphate (0.1 M, pH 7.2) buffered 2.5% glutaraldehyde, immediately after they were removed from incubation bottles, for fixation of presumed biofilm. After 2–16 h the coupons were rinsed with phosphate buffer three times. Dehydration was carried out with an ethanol series, followed by hexamethyldisilazane (Fluka, Switzerland). The membrane was coated with Au/Pd (10 nm, 208 HR High Resolution Sputter Coater, Cressington Scientific Instruments Inc., USA) and examined with Hitachi S-4800 FESEM (Japan) operated at 1–5 kV.

## Results

### Water Chemistry

The groundwater from 100 m depth from a borehole in the VLJ-repository in Olkiluoto was brackish and sulfate rich (**Table 2**). The water was slightly alkaline with pH of 7.8 measured on site. Conductivity (Ec) of the groundwater was 2.11 mS cm^-1^. After the 3 months (3M) incubation period the conductivity of the water in the incubation bottles slightly decreased to 2.07 at RT (3M-RT) and 1.91 at 6°C (3M-6°C). However, after 8 months (8M) the conductivity increased to 2.95 and 2.97 in the RT and 6°C incubations, respectively (**Table 3**). The redox potential (mV vs. standard hydrogen electrode, SHE) was -78.7 in 3M-RT and -42.8 in 3M-6°C. After 8 months the redox potential was -78.3 mV vs. SHE and -77.7 in the 8M-RT and 8M-6°C microcosms, respectively. The pH also increased during the experiment period to 8.2 and 8.4 in the microcosms at 6°C and room temperature, respectively (**Table 3**).

**Table 2:** The main chemical components in the groundwater in the beginning of the experiment.

Al μg L^-1^	Br mg L^-1^	Ca mg L^-1^	Cl mg L^-1^	Fe mg L^-1^	Mg mg L^-1^	Mn mg L^-1^	K mg L^-1^	SiO_2_ mg L^-1^	Na mg L^-1^	SO_4_ mg L^-1^	pH	Ec mS cm^-1^
4	1.4	57	430	0.08	17	0.12	7.7	14	354	123	7.8	2.11

**Table 3:** Characteristics of water and surface layers.

	pH	Conductivity (mS cm^-1^)	Redox potential (mV vs. SHE)	Deposits main components (w%)	Corrosion rate μm a^-1^
Groundwater in the beginning	7.8	2.11		–	–
3M-RT	8.2	2.07 ± 0.006	-78.7 ± 3.7	60.9 Fe, 10.4 C, 31.4 O, 0.2 S	1.4
3M-6°C	8.1	1.91 ± 0.06	-42.8 ± 0	62.7 Fe, 9.4 C, 14.9 O, 14.1 S	11.4
8M-RT	8.4	2.95 ± 0	-77.7 ± 37.6	76.5 Fe, 2.2 C, 15.6 O, 8.2 S	1.4
8M-6°C	8.2	2.97 ± 0.02	-78.3 ± 12.8	91.2 Fe, 2.2 C, 13.2 O, 0.5 S	–

### Microbial Community Analyses

**FIGURE 1:**
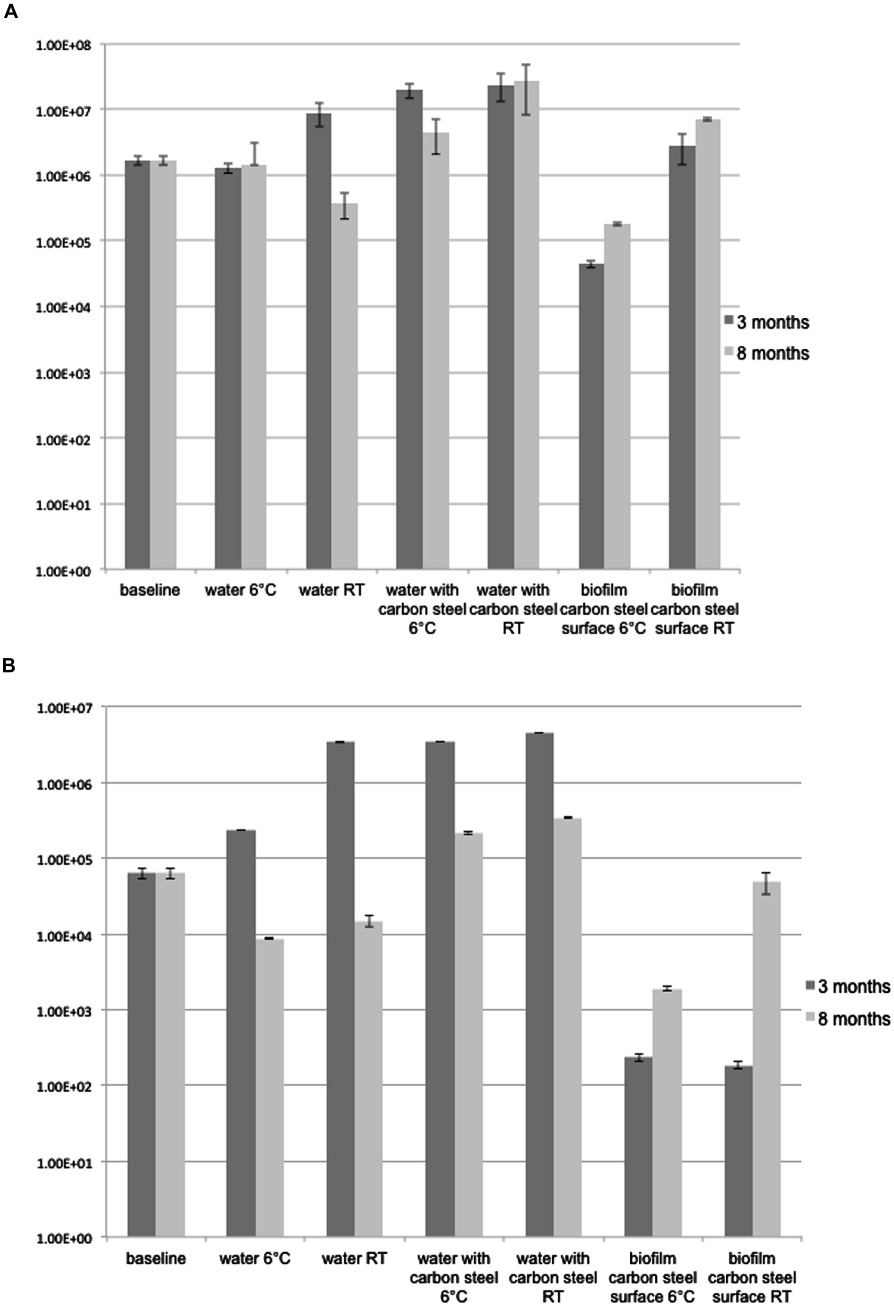
**(A)** 16S rRNA gene copies ml^-1^ groundwater or carbon steel (cm^2^)^-1^. The error bars present SE of mean (*n* = 3). **(B)** *dsr*B gene copies ml^-1^ groundwater or carbon steel (cm^2^)^-1^. The error bars present SE of mean (*n* = 3).

The number of bacterial 16S rRNA genes in the groundwater was 1.69 × 10^6^ copies mL^-1^ at the beginning of the experiment. After 3-months a 10-fold increase in the concentration of bacterial 16S rRNA genes was detected (1.95 × 10^7^ copies mL^-1^ in 3M-6°C, and 2.23 × 10^7^ mL^-1^ in 3M-RT, *p* < 0.01) in the groundwater exposed to carbon steel (**Figure 1A**). After 8 months the concentrations of 16S rRNA gene copies were 4.53 × 10^6^ mL^-1^ in 8M-6°C and 2.76 × 10^7^ mL^-1^ in 8M-RT (**Figure 1A**). The number of 16S rRNA gene copies from groundwater without carbon steel at 6°C remained at the same level as in the beginning of the experiment, i.e., 1.29 × 10^6^ mL^-1^ in 3M-6°C and 1.43 × 10^6^ mL^-1^ in 8M-6°C, *p* < 0.01 (**Figure 1A**). However, at room temperature the concentration of 16S rRNA gene copies had increased to 8.81 × 10^6^ copies mL^-1^ in 3M-RT compared to that of the baseline samples but decreased again toward 8 month of incubation to 3.81 × 10^5^ copies mL^-1^ in 8M-RT (**Figure 1A**). A denser biofilm, according to the number of 16S rRNA gene copies, was detected on RT-coupons (2.82 × 10^6^ and 7.03 × 10^6^ copies cm^-2^, after 3 and 8 months, respectively, *p* < 0.05) than on 6°C-coupons (4.42 × 10^4^ and 1.77 × 10^5^ copies cm^-2^, after 3 and 8 months, respectively, *p* < 0.05; **Figure 1A**).

**FIGURE 2:**
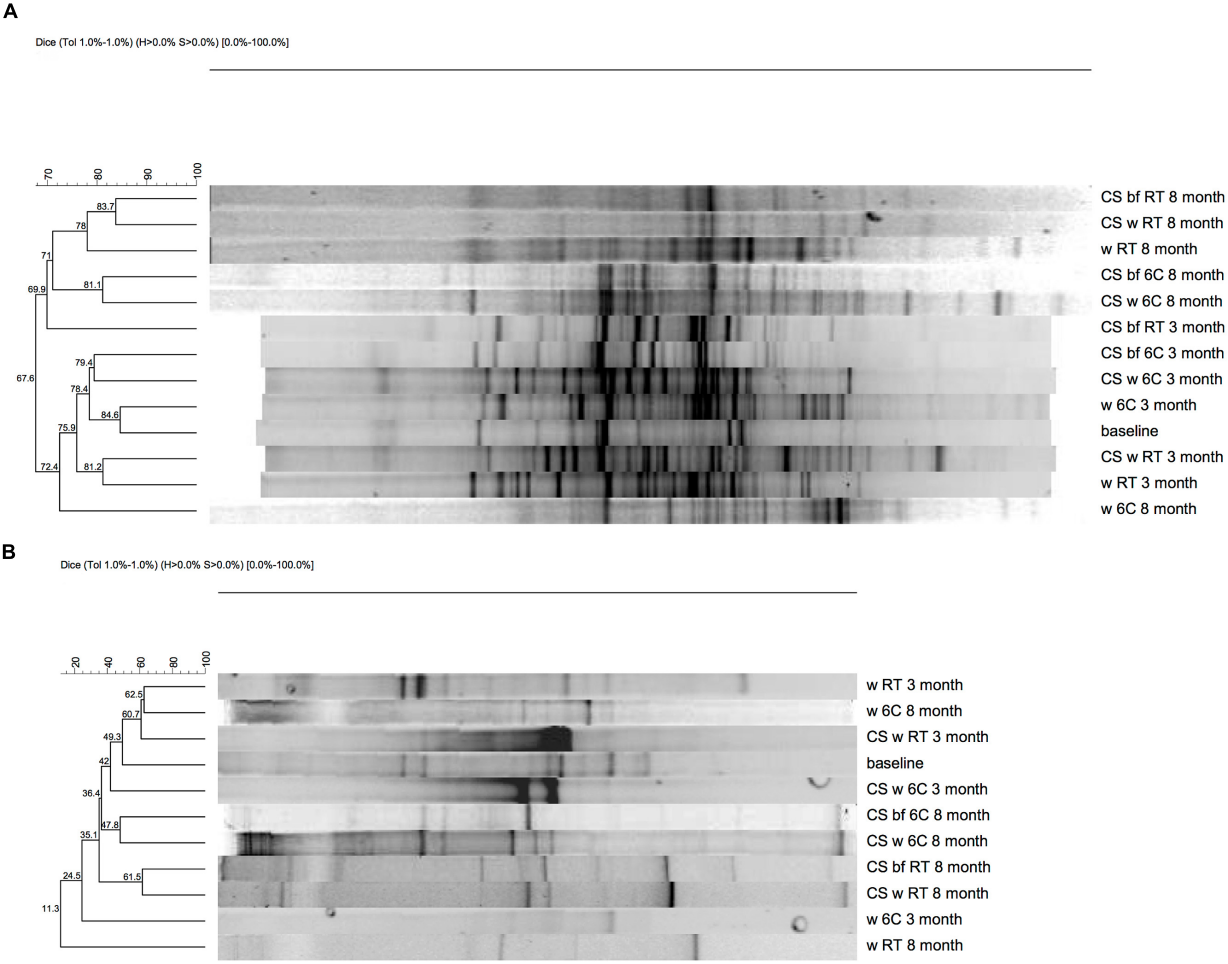
**Denaturing gradient gel electrophoresis (DGGE), **(A)** 16S rRNA **(B)***dsr*B gene**.

The DGGE analysis of the bacterial community showed a distinct and diverse banding pattern in the groundwater samples at the beginning of the experiment (**Figure 2A**). The bacterial 16S rRNA gene profiles showed between 4 and 35 different bands per sample, after the 3-months incubation period (**Figure 2A**). DGGE analysis revealed between 7 and 26 bands after the 8-months incubation period (**Figure 2A**). The diversity of DGGE bands increased notably compared to the baseline samples over the 3-months incubation period (**Figure 2A**). DGGE analysis showed that the bacterial species composition of the biofilm formed on carbon steel coupons was diverse and bacterial communities of the incubation water and the biofilms of the corresponding carbon steel samples were generally similar, with exception of the water and biofilm of the microcosms at room temperature after 3-months. It was also shown that the bacterial diversity was greater in the groundwater exposed to carbon steel compared to the groundwater without carbon steel (**Figure 2A**).

**FIGURE 3:**
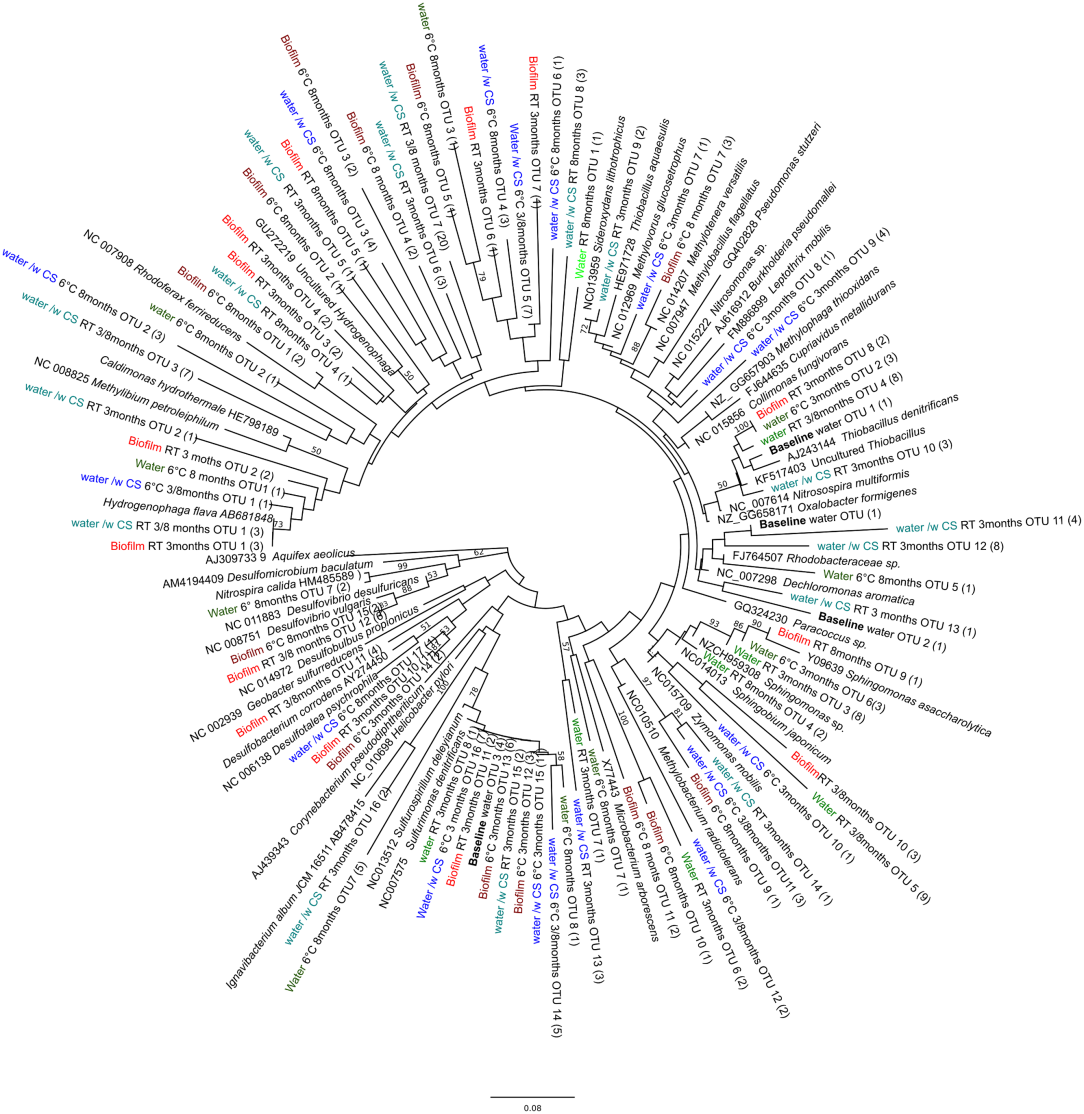
**Phylogenetic tree of bacterial diversity, based on the 16S rRNA gene sequences obtained by 16S gene-based PCR-DGGE, in relation to cultured- and the closest uncultured relatives.** Parentheses indicate the number of individual sequences within the OTU. Bootstrap values, calculated from 1,000 repetitions, are shown at branch points with >50% support. The scale bar indicates 0.08 nucleic acid substitutions. The tree is rooted by *Aquifex aeolicus.*

The obtained 16S rRNA gene sequences fell into four different bacterial clusters in the phylogenetic analysis, Betaproteobacteria, Alphaproteobacteria, Epsilonroteobacteria, and Deltaroteobacteria (**Figure 3**). The dominating class was Betaproteobacteria (**Figure 3**). In the original groundwater, only sequences belonging to Betaproteobacteria were detected (**Figure 3**).

### The Composition of the SRB Community

The number of *dsr*B gene copies mL^-1^ analyzed by qPCR was 6.3 × 10^4^ in the original groundwater. After 3-months at 6°C the concentration increased to 2.3 × 10^6^ copies mL^-1^ in the water (*p* < 0.01), but decreased to 8.7 × 10^3^ copies mL^-1^ at the end of the 8 months of incubation at 6°C (*p* < 0.01; **Figure 1B**). At RT the detected amounts of *dsr*B copies were slightly higher, 3.4 × 10^6^ and 1.5 × 10^4^ copies mL^-1^ after 3 and 8 months incubation, respectively (**Figure 1B**). When the groundwater was exposed to carbon steel the amount of *dsr*B copies in water increased to 3.5 × 10^6^ mL^-1^ after 3 months in both temperatures (*p* < 0.01) and decreased to the level of 2–3 × 10^5^ copies mL^-1^ after 8 months (**Figure 1B**). The amount of *dsr*B gene copies in biofilm on the 3M-6°C-coupons and 3M-RT-coupons was 1.9 × 10^2^ and 2.3 × 10^2^ copies cm^-2^, respectively (**Figure 1B**). After 8-months the amount of *dsr*B in biofilm increased to 5 × 10^4^ and 1.9 × 10^3^ copies cm^-2^ on 8M-6°C-coupons and 8M-RT-coupons, respectively (*p* < 0.01; **Figure 1B**).

**FIGURE 4:**
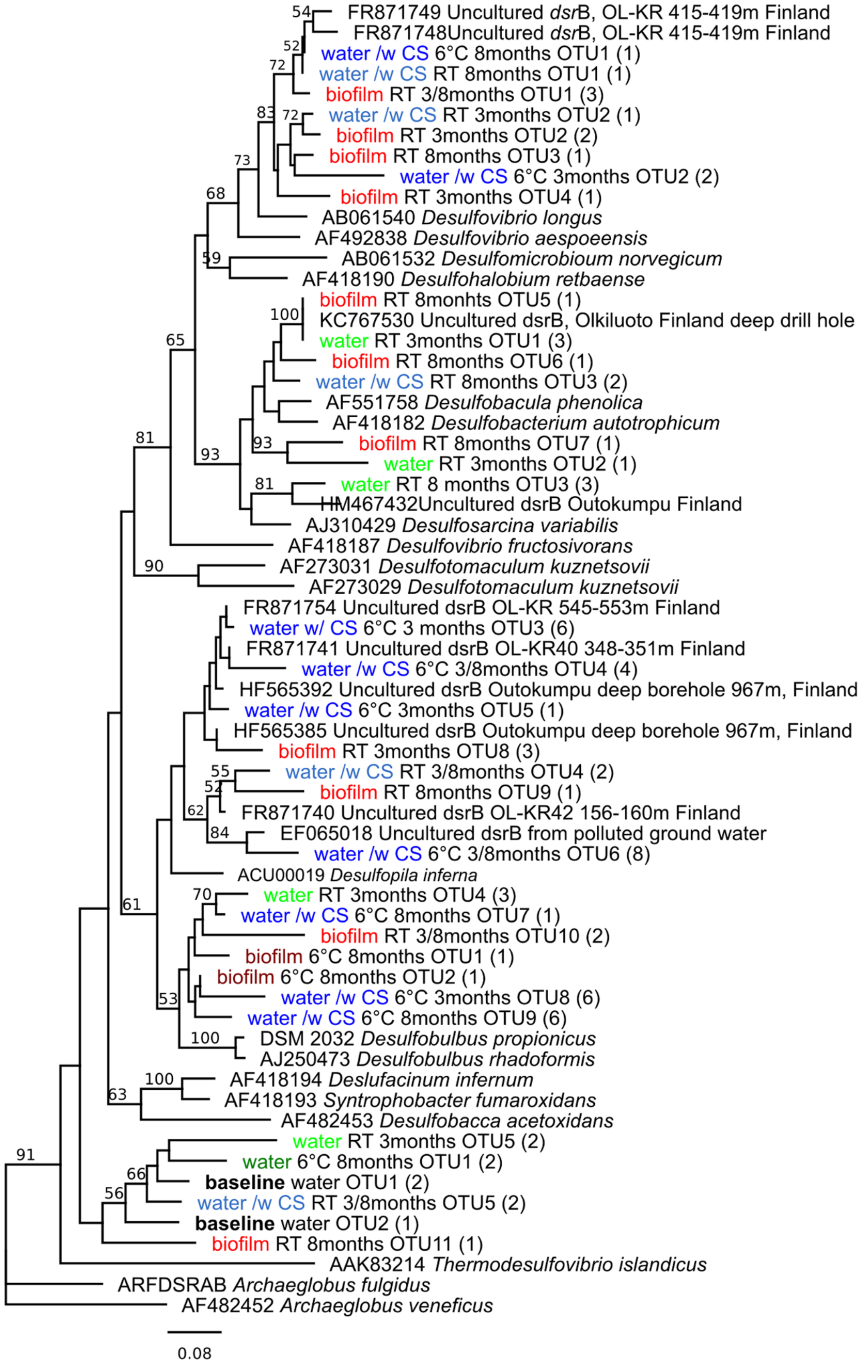
**Phylogenetic tree of sulfate reducing bacteria, based on the *dsr*B sequences (amino acid) obtained by *dsr*B gene-based PCR-DGGE, in relation to cultured SRB and the closest uncultured relatives. Parentheses indicate the number of individual sequences within the OTU. Bootstrap values, calculated from 1,000 repetitions, are shown at branch points with >50% support. The scale bar indicates 0.08 amino acid substitutions. The tree is rooted by *Archaeoglobus veneficus***.

In all samples, the PCR-DGGE profiles revealed diverse SRB communities with several different *dsrB* amplicons. In the original groundwater three DGGE bands were detected belonging to genus *Thermodesulfovibrio* (**Figure 4**). Between 7 and 13 *dsr*B-gene DGGE bands were observed per sample after 3-months and between 7 and 17 bands after 8-months of incubation (**Figure 2B**). The diversity of the SRB community had changed over the 3-months incubation at 6°C when bands belonging to families Desulfovibrionaceae, Desulfobulbaceae, and Thermodesulfovibrio were detected in the water. At RT the detected bands belonged to families Desulfobacteraceae, Desulfovibrionaceae, Desulfobulbaceae, and Thermodesulfovibrio. All *dsrB* amplicons detected belonged to Deltaproteobacteria and affiliated with the families Desulfobacteraceae, Desulfovibrionaceae, Desulfobulbaceae and Thermodesulfovibrio (**Figure 4**). Similar *dsr*B-genes were detected both from the biofilm on the carbon steel and from the water (**Figures 2B** and **4**). The majority of all sequences resembled *dsrB* genes of the family Desulfobulbaceae. SRB belonging to the genus *Desulfobacula* were detected only from samples that were incubated at room temperature (**Figure 4**).

### Corrosion

**FIGURE 5:**
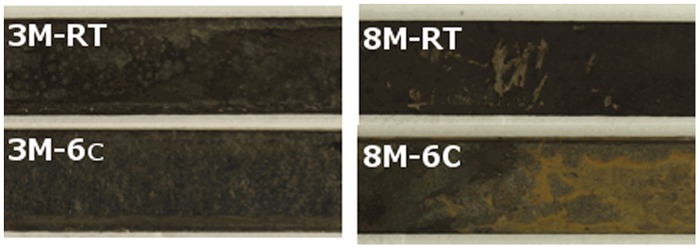
**Surface of carbon steel**.

After 3 months of exposure to groundwater a small amount of dark deposit could be seen on the surfaces of carbon steel coupons (**Figure 5**) at both temperatures. By visual examination, the carbon steel coupons were covered by a thicker, more even and darker deposit layer when incubated at room temperature (3M-RT-coupons) compared to those kept at 6°C (3M-6°C-coupons; **Figure 5**). In addition, the 3M-6°C-coupons had light brown areas on the surfaces, which were not detected on the surfaces of the 3M-RT-coupons (**Figure 5**). A higher concentration of sulfur was detected in the deposit on the 3M-RT-coupons compared to the 3M-6°C-coupons (14.1 and 0.2 w-%, respectively; **Table 3**). During 8 months (8M) the formation of the deposits became more intensive, the deposits were darker and contained more iron (w-% 76.5 RT/ 91.2 6°C) but less oxygen (w-% 15.6 RT/ 13.2 6°C) and carbon (w-% 2.2 RT/6°C) on the surfaces both 8M-6°C-coupons and 8M-RT-coupons compared to 3-months exposure (**Figure 5**; **Table 3**). In addition, the concentration of sulfur was higher on the 8M-RT-coupons compared to the 8M-6°C-coupons (8.2 w-% and 0.5 w-%, respectively).

**FIGURE 6:**
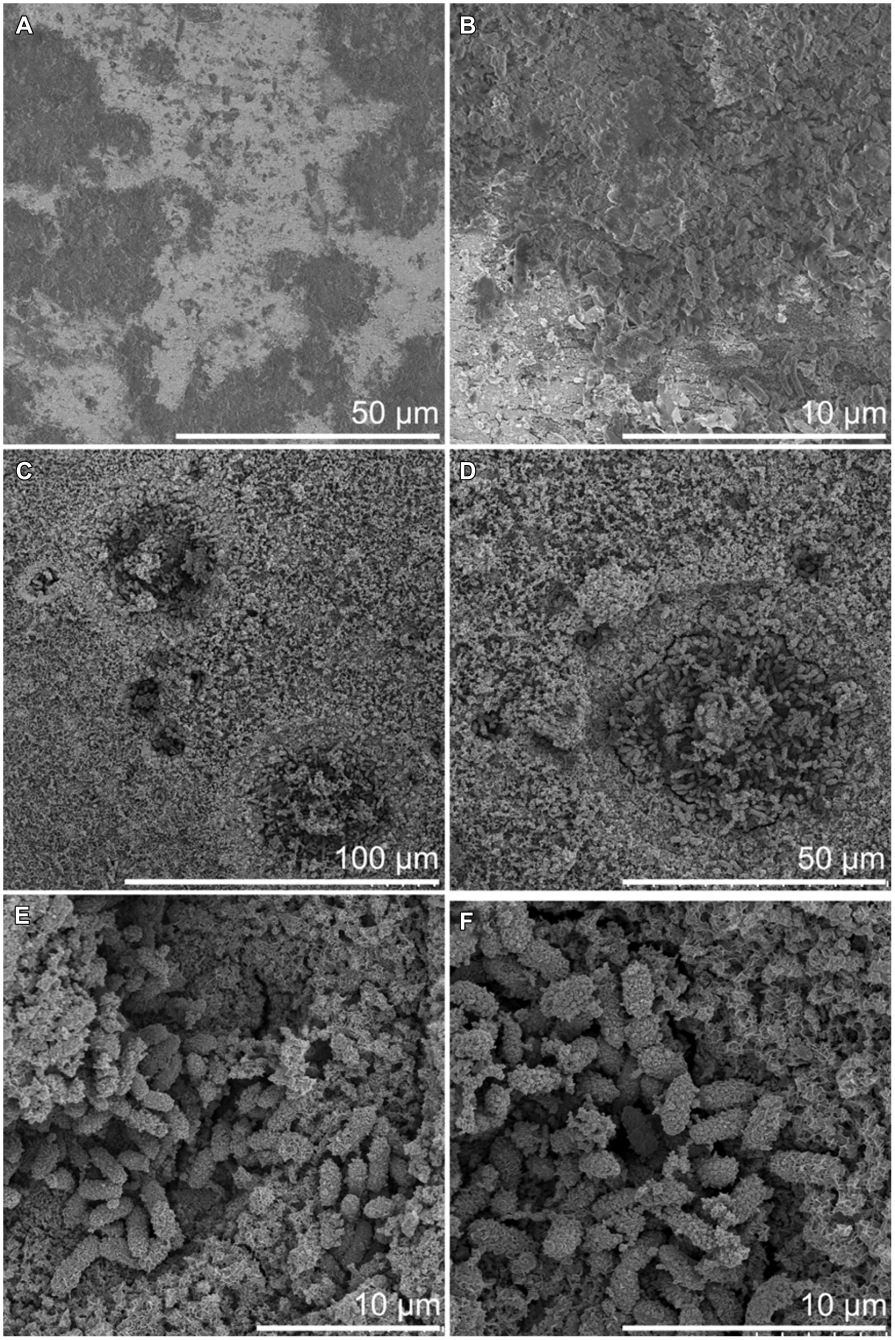
**Surface of carbon steel incubated at 6°C by FE-SEM. (A,B)** After 3 months incubation. **(C–F)** After 8 month incubation.

**FIGURE 7:**
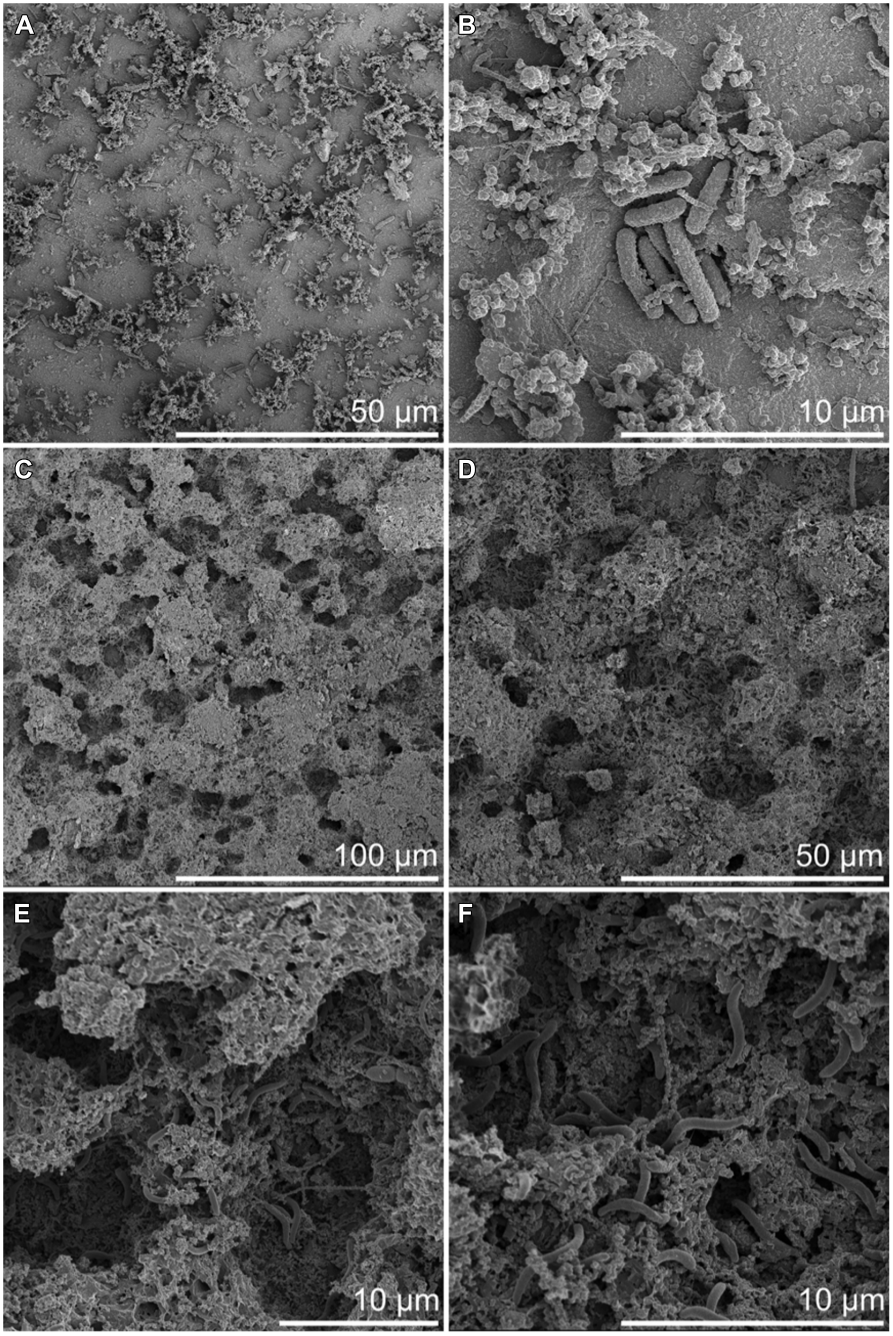
**Surface of carbon steel incubated at RT by FE-SEM. (A,B)** After 3 months incubation. **(C–F)** After 8 months incubation.

After 3 months of exposure, measurable corrosion rates of 1.4 and 11.4 μm a^-1^ were detected on 3M-RT-coupon and one 3M-6°C-coupon, respectively. After 8 months of exposure measurable corrosion of 1.35 μm a^-1^ was detected in one 3M-RT-coupon. In addition, incipient localized corrosion was observed by stereomicroscope and FE-SEM examination in all coupons after pickling (**Figures 6** and **7**). The 8M-RT-coupon, having the measurable corrosion rate, also presented shallow corrosion pits with a maximum diameter of 200 μm (**Figure 7**). Also, a small number of smaller corrosion pits with a diameter of 50 μm were observed on one 8M-6°C-coupon (**Figure 6**).

Besides the corrosion pits, the FE-SEM examination revealed microorganisms adhered to the carbon steel surfaces. Microorganisms were detected on the steel surface already after 3-months but the corrosion pits evolved toward the end of the incubation period and were visible in samples incubated for 8 months (**Figures 6** and **7**). A higher number of microorganisms were detected on the surface of the 3M-RT-coupons than on the 3M-6°C-coupons. After 8 months of incubation a dense biofilm as well as a substantial number of corrosion pits had formed on the carbon steel coupons incubated at both temperatures. Temperature also affected the type of microorganisms predominating on the carbon steel surfaces (**Figures 6** and **7**). In the pits formed at 6°C microbial cells were mostly 2 μm × 1 μm rods covered with corrosion products (**Figure 6**), whereas at RT the pits were covered with long spirilli-formed microbial cells (**Figure 7**).

## Discussion

Microbially induced corrosion and its effects on metallic materials are of critical importance globally. For example, the annual cost of corrosion problems caused by MIC is estimated to be 1 trillion USD worldwide (Fleming, 1996). The biofilm formation is the key step in inducing MIC (Lee and Characklis, 1993). Corrosion accelerating conditions, especially localized corrosion, can evolve rapidly under the biofilm. In the present study, it was demonstrated that microorganisms in natural deep groundwater have great affinity to form biofilm on the surface of carbon steel and cause localized corrosion even when corrosion cannot be detected by gravimetric analysis. Our results indicate that deep groundwater microorganisms benefited from carbon steel. In the presence of carbon steel the planktonic microbial community in the incubation water was more diverse and 100-fold more abundant compared to the environment without carbon steel.

The Betaproteobacteria were the most dominant bacterial class in all samples where carbon steel was present, whereas in groundwater incubated without carbon steel the microbial community was less diverse and resembled more the community detected from groundwater in the beginning of the experiment. Betaproteobacteria belonging to Burkholderiales and Hydrogenophilales are known to use hydrogen as an energy source (Willems et al., 1989; Stöhr et al., 2001). Hydrogen is released at the cathodic site, i.e., from the carbon steel, in corrosion processes and thus may attract hydrogenotrophic Betaproteobacteria (Cord-Ruwisch and Widdel, 1986). Betaproteobacteria also belonging to Burkholderiales, on the other hand, are able to oxidize iron, which has been shown to accelerate corrosion of iron (Hedrich et al., 2011; Liu et al., 2012). In our study the dominant iron oxidizing Betaproteobacteria appeared to be temperature dependent, showing *Leptothrix* species in the samples incubated at 6°C. At RT the dominant iron oxidizing Betaproteobacteria resembled most closely *Sideroxydans* species. The reddish corrosion deposit formed on surfaces of carbon steel is likely to be a result from iron oxidizing bacteria. Iron reducing Betaproteobacteria were detected in samples where carbon steel was available. These iron reducers may cause corrosion by the reduction and removal of passive films of ferric compounds under anoxic conditions whereas under oxic conditions they are able to protect carbon steel from corrosion by improving the surface passivation (Herrera and Videla, 2009).

Sulfate-reducing bacteria are generally assumed to be the main bacterial group causing MIC in anoxic environments (Hamilton, 1985; Lee et al., 1995). SRB produce hydrogen sulfide, which is a corrosive agent. They are also believed to consume the excess hydrogen and thus stimulate the corrosion reaction. Nevertheless, an alternative reaction has recently been reported by which microbes can induce the corrosion of steels by using elemental iron (Fe^0^) as electron source (Dinh et al., 2004). This process is used by at least some SRB and methanogenic archaea. In nutrient-poor environments, such as the deep groundwater examined in this study, the microbes may be more “aggressive” toward steels, since the concentration of organic electron donors is low (Xu and Gu, 2011). In the native groundwater at the beginning of our experiment, only *dsr*B sequences belonging to the Thermodesulfovibrio family were detected. However, in the presence of carbon steel the diversity of the SRB community was increased to comprise Deltaproteobacterial SRB. The SRB were detected from both the incubation water and as part of the biofilm attached to the surfaces of carbon steel. The planktonic and biofilm SRB communities were similar but were affected by temperature. At RT SRB belonging to the family Desulfobacteraceae were detected whereas at 6°C the *dsr*B genes belonged to family Desulfobulbaceae. The Desulfobulbaceae include Fe^0^-utilizing SRB, such as the *Desulfopila inferna.* In our experiment, this bacterium was abundant in water exposed to carbon steel, and it has been proposed that *Desulfopila inferna* is capable of using Fe^0^ as electron donor for sulfate reduction (Dinh et al., 2004; Enning et al., 2012). In addition, *dsr*B sequences matching *Desulfobacterium corrodens*, another Fe^0^ utilizing SRB, were found in biofilm at RT (Enning et al., 2012). Enning et al. (2012) reported that the corrosion crust observed together with these SRB consisted of Fe, S, O, and C. This is in accordance with our results, where these compounds were also detected, especially after the 3-months incubation period. Both the SRB community and the corrosion products were different depending on the temperature of incubation. At RT the amount of sulfur in deposits was 14 times higher than at 6°C while the abundance of Si was higher at 6°C than at RT. However, the amount of Fe in the deposits was not affected by temperature and was elevated in all conditions after 8-months of incubation. Microbial cells in the samples incubated at room temperature were typically long (up to 4 μm), slender (0.3 μm) and had curved or corkscrewed shape. Cells had very smooth cell envelope suggesting that they may be Gram-positive. In addition, stalk-like structures, formed customarily by iron oxidizing Proteobacteria were clearly visible at RT. At 6°C microbes were rod shaped and frequently observed as diplobacilli and had an appearance suggesting that they are Gram-negative bacteria. According to SEM images, the incubation temperature had significant effect on the morphology of the microorganisms attached on the surfaces of the samples. In conclusion, our results demonstrate that in order to obtain reliable results on MIC, the measurements should be carried under *in situ* temperature. In addition, the carbon steel had a positive effect on the bacterial community size and composition, indicating that the native groundwater bacterial communities may benefit from compounds in carbon steel and that the corrosion of carbon steel and subsequent release of these compounds may cause the number of microorganisms to increase and composition of microbial community to change.

## Author Contributions

PR, LC, MV, MB conceived, designed and performed the experiments, analyzed the data and wrote the paper. MR performed the SEM analyses, analyzed the data and wrote the paper. ES analyzed the DGGE data and wrote the paper.

## Conflict of Interest Statement

The authors declare that the research was conducted in the absence of any commercial or financial relationships that could be construed as a potential conflict of interest.
